# Add-On Effect of Probucol in Atherosclerotic, Cholesterol-Fed Rabbits Treated with Atorvastatin

**DOI:** 10.1371/journal.pone.0096929

**Published:** 2014-05-08

**Authors:** Yuka Keyamura, Chifumi Nagano, Masayuki Kohashi, Manabu Niimi, Masanori Nozako, Takashi Koyama, Reiko Yasufuku, Ayako Imaizumi, Hiroyuki Itabe, Tomohiro Yoshikawa

**Affiliations:** 1 Free Radical Research Project, Otsuka Pharmaceutical Co., Ltd., Tokushima, Japan; 2 Division of Biological Chemistry, Department of Molecular Biology, Showa University School of Pharmacy, Tokyo, Japan; Max-Delbrück Center for Molecular Medicine (MDC), Germany

## Abstract

**Objective:**

Lowering the blood concentration of low-density lipoprotein (LDL) cholesterol is the primary strategy employed in treating atherosclerotic disorders; however, most commonly prescribed statins prevent cardiovascular events in just 30% to 40% of treated patients. Therefore, additional treatment is required for patients in whom statins have been ineffective. In this study of atherosclerosis in rabbits, we examined the effect of probucol, a lipid-lowering drug with potent antioxidative effects, added to treatment with atorvastatin.

**Methods and Results:**

Atherosclerosis was induced by feeding rabbits chow containing 0.5% cholesterol for 8 weeks. Probucol 0.1%, atorvastatin 0.001%, and atorvastatin 0.003% were administered solely or in combination for 6 weeks, beginning 2 weeks after the start of atherosclerosis induction. Atorvastatin decreased the plasma concentration of non-high-density lipoprotein cholesterol (non-HDLC) dose-dependently; atorvastatin 0.003% decreased the plasma concentration of non-HDLC by 25% and the area of atherosclerotic lesions by 21%. Probucol decreased the plasma concentration of non-HDLC to the same extent as atorvastatin (i.e., by 22%) and the area of atherosclerotic lesions by 41%. Probucol with 0.003% atorvastatin decreased the plasma concentration of non-HDLC by 38% and the area of atherosclerotic lesions by 61%. Co-administration of probucol with atorvastatin did not affect the antioxidative effects of probucol, which were not evident on treatment with atorvastatin alone, such as prevention of in vitro LDL-oxidation, increase in paraoxonase-1 activity of HDL, and decreases in plasma and plaque levels of oxidized-LDL in vivo.

**Conclusions:**

Probucol has significant add-on anti-atherosclerotic effects when combined with atorvastatin treatment; suggesting that this combination might be beneficial for treatment of atherosclerosis.

## Introduction

Atherosclerosis is the root cause of cardiovascular diseases such as myocardial infarction, stroke, and peripheral vascular disease, all of which are present in most human populations. Therefore, it remains a leading cause of mortality despite substantial therapeutic progress due to the widespread use of statins, which inhibit 3-hydroxy-3-methylglutaryl-coenzyme A (HMG-CoA) reductase, a key enzyme in cholesterol biosynthesis. Large-scale clinical trials using statins for both primary and secondary prevention have shown a significant reduction in cardiovascular events, mainly due to the lowering of plasma concentrations of low-density lipoprotein (LDL) cholesterol; however, the capacity of statins to prevent cardiovascular events is still limited to 30% to 40% of the patients treated even when intensive statin therapy is used [1], [2]. To lower the plasma concentration of LDL cholesterol sufficiently, some combinational therapies such as statin plus ezetimibe have been tested; however, it remains unclear whether the clinical efficacy of combination therapy is better than that of statin monotherapy [3]. Therefore, new drugs or strategies are sorely needed to reduce the number of events in patients who have an inadequate response to statin treatment.

Atherosclerosis results from multiple complex processes involving injurious stimuli and responses of the arterial wall to these stimuli. Such events occur in a hyperlipidemic environment. Oxidized plasma lipoproteins produced by free radical peroxidation of lipids in such processes play a pivotal role; the cytotoxic action of oxidized plasma lipoproteins damages the endothelium, induces inflammatory responses such as enhanced synthesis of inflammatory cytokines and chemokines in endothelial cells, and leads to foam cell formation [4]–[6]. Administration of antioxidants with hypolipidemic drugs is, therefore, a rational means to prevent the development of atherosclerosis.

Probucol, a lipid-lowering drug that has potent antioxidative activities, prevents oxidation of LDL [7] and attenuates atherosclerotic lesion development in most animal models [8]–[10] with the exception of apolipoprotein E -deficient mice [11]. Partly because of a consistent reduction in serum high-density lipoprotein cholesterol (HDLC) levels and possible QT interval prolongation with no obvious effect on changes in the luminal diameter of femoral arteries [12], probucol was withdrawn from use in most Western countries after statins appeared [13]. However, probucol has still been used clinically in Asian countries such as Japan, China, and Korea. Moreover, it has been shown to reduce the intima-media thickness of the carotid artery and the incidence of cardiovascular events in patients with asymptomatic hypercholesterolemia [14], prevent secondary cardiovascular events in patients with heterozygous familial hypercholesterolemia [15], and reduce the risk of all-cause mortality in patients with coronary artery disease and a history of complete revascularization [16].

Given that statins have a strong cholesterol-lowering effect and probucol has strong antioxidative activities, it can be inferred that adding probucol to statin treatment might be beneficial in the treatment of atherosclerosis in patients who have an inadequate response to statins. Several studies in animals [17] and in humans [18] have examined the effects of probucol combined with statins on atherosclerosis; however, these reports did not compare the effect of combined therapy to that of monotherapy. Both drugs also possess other anti-atherogenic effects such as antiinflammatory effects [13], [19]; therefore, it remains unclear whether probucol combined with statins would have a significant add-on effect in the treatment of atherosclerosis without interfering with the pleiotropic effects of these drugs.

In this study, the add-on effects of probucol in the treatment of atherosclerosis when combined with atorvastatin were evaluated in a representative animal model of atherosclerosis, 0.5% cholesterol-fed rabbits, because the lipid metabolism and pathologic characteristics of atherosclerosis in these animals are closer to those of humans than those of mice are.

## Materials and Methods

### Ethics Statement

This study was carried out in strict accordance with the Guidelines for the Animal Care and Use of the Otsuka Pharmaceutical Co., Ltd. which conforms to the international norms stipulated by the Ministry of Health, Labour and Welfare in Japan. The protocol was approved by the Institutional Animal Care and Use Committee of the Otsuka Pharmaceutical Co., Ltd. (Permit Number: 11-0221). All efforts were made to maximize animal welfare and minimize suffering.

### Materials

Probucol was provided by Otsuka Pharmaceutical Co., Ltd. (Tokushima, Japan), and atorvastatin calcium was purchased from Sequoia Research Products Ltd. (Pangbourne, United Kingdom).

### Animals

New Zealand White rabbits (specific pathogen-free, 6–7 weeks old) were purchased from Kitayama Labes Co., Ltd. (Nagano, Japan). The rabbits were acclimatized and fed 80 g/day of cholesterol-enriched diet (HC-RC4) that contained 0.5% (wt/wt) cholesterol in a standard rabbit feed, RC4 (Oriental Yeast Co., Ltd., Osaka, Japan), for 2 weeks, and housed in separate cages in animal rooms where environmental controls were set to maintain the following conditions: temperature, 23±2°C; humidity, 60±10%. Body weights and plasma parameters were measured at 1 and 2 weeks after the start of cholesterol loading. Some rabbits were excluded before allocation because of their very high (>1200 mg/dL) or low (<770 mg/dL) plasma total cholesterol (TC) concentrations or high alanine aminotransferase (ALT) values (>100 IU/L). The remaining rabbits were allocated to 6 groups based on plasma TC concentrations at 2 weeks and on the change in TC concentration at 1 and 2 weeks as follows: group 1, control (n = 11); group 2, 0.1% probucol (n = 11); group 3, 0.001% atorvastatin (n = 11); group 4, 0.001% atorvastatin +0.1% probucol (n = 11); group 5, 0.003% atorvastatin (n = 12); group 6, 0.003% atorvastatin +0.1% probucol (n = 12). Random allocation was performed by automated assignment using SAS software (Release 9.1, SAS Institute Inc., Tokyo, Japan). After allocation, the rabbits were fed 80 g/day of HC-RC4 mixed with or without the drugs for 6 weeks. We determined the doses of the drugs based on the data in in-house preliminary studies: the doses of atorvastatin were shown to have significant plasma TC-lowering effects but to slightly increase (0.003%) or not increase (0.001%) levels of plasma markers of liver damage, which are equivalent to the maximal dose prescribed in a clinical setting; the dose of probucol (0.1%) was the minimum dose that caused a significant reduction in atherosclerotic lesion formation at lower plasma concentrations of probucol than were seen in a clinical study [7]. We believe that the doses of these drugs mimic their clinical usage in order to explore a new strategy for atherosclerosis treatment that augments the limited anti-atherosclerotic effects of statins. All diets were prepared by Oriental Yeast Co., Ltd. (Osaka, Japan). Body weights and plasma parameters were measured at 2, 4, and 6 weeks after the first administration.

### Measurement of Plasma Lipids and Biochemical Markers

Blood was drawn from the ear vein and collected into a heparinized syringe without anesthesia to measure the plasma lipid level, levels of biochemical markers, and probucol concentration; blood was also collected from the inferior vena cava into a heparinized syringe to measure plasma parameters (levels of C-reactive protein [CRP], paraoxonase-1 [PON-1], and oxidized lipids) and into an EDTA-coated syringe under pentobarbital anesthesia to isolate plasma lipoproteins.

Plasma lipids (TC, HDLC, phospholipids, and triglyceride) and blood biochemical markers (aspartate aminotransferase [AST] and ALT) were measured using reagents by Wako Pure Chemical Co., Ltd. (Osaka, Japan). Non-HDLC concentration was calculated by subtracting the HDLC concentration from TC concentration. Platelet activating factor acetylhydrolase (PAF-AH) activity and the lysophosphatidylcholine (LysoPC) content in lipoprotein fractions were measured using an AZWELL Auto PAF-AH kit and AZWELL LPC Assay kit (Alfresa Pharma Corporation, Osaka, Japan), respectively. PAF-AH activity and the LysoPC content per mL in lipoprotein fractions were divided by their protein concentrations to calculate the PAF-AH activity and LysoPC content relative to protein concentrations in the lipoproteins. Plasma PON-1 activity evaluated as paraoxonase, arylesterase, and homocysteine thiolactonase activities per mL were measured using Paraoxonase Assay kit (Nikken Seil Co., Ltd., Shizuoka, Japan), Arylesterase Assay kit (Nikken Seil Co., Ltd., Shizuoka, Japan), and Alfresa Auto Assay kit (Alfresa Pharma Corporation, Osaka, Japan), respectively, and was then divided by the protein concentration of HDL to calculate the PON-1 activity relative to HDL protein. Plasma CRP concentration was measured using Rabbit CRP ELISA kit (Shibayagi Co., Ltd., Gunma, Japan). The plasma concentration of probucol was measured using a lipid chromatograph/tandem mass spectrometer by Mitsubishi Chemical Medience Corporation (Tokyo, Japan).

### Isolation of Plasma Lipoproteins

Plasma very-low-density lipoprotein (VLDL), LDL, and HDL were separated by the method of Hatch and Lee [20]. This method utilizes stepwise density-gradient ultracentrifugation. Briefly, chylomicrons were removed as the upper fraction (d = 1.006) from the bottom fraction (d = 1.024) of plasma by centrifugation at 5×10^4^ *g* for 20 minutes at 16°C. VLDL was separated as the upper fraction (d = 1.006) from the bottom fraction by centrifugation at 2×10^5^ *g* for 12 hours at 16°C. LDL was separated as an upper fraction (d = 1.006–1.063) after adjustment of the bottom fraction density to 1.063 and centrifugation at 2×10^5^ *g* for 14 hours at 16°C. HDL was separated as the upper fraction (d = 1.063–1.21) after adjustment of the bottom fraction density to 1.21 and centrifugation at 2×10^5^ *g* for 27.5 hours at 16°C. The densities were adjusted with potassium bromide (KBr) solution. To remove KBr or KBr and EDTA, the separated lipoproteins were dialyzed extensively against 0.25 mM EDTA-4 Na (pH 7.4)/Dulbecco’s phosphate buffer solution (PBS) or PBS, respectively, in a refrigerator under protection from light. Protein concentrations of the lipoproteins were measured by a modified Lowry method (*DC* Protein Assay by Bio-Rad Laboratories, Inc, Tokyo, Japan), and the purities of the lipoprotein fractions were checked by SDS-polyacrylamide gel electrophoresis followed by protein staining with Coomassie brilliant blue.

### Measurement of Plasma Oxidized LDL Level

The plasma oxidized LDL (OxLDL) level was measured by a modified sandwich enzyme-linked immunosorbent assay (ELISA) using LDL dialyzed against PBS and a monoclonal antibody against OxLDL (DLH3 [21]) [22]. Briefly, blocking solution (Tris-buffered saline [TBS] with 1% bovine serum albumin), washing buffer (TBS with 0.2% polysorbate 20), antibody dilution buffer (TBS with 2% skim milk [Morinaga Milk Industry Co., Ltd., Tokyo, Japan]), and substrate solution (an aqueous solution of 9.7% diethanolamine, 0.02% sodium azide, and 0.01% magnesium chloride, adjusted to pH 9.8 with hydrochloric acid) were made up. Standard solutions were prepared as follows: pooled LDL separated from the plasma of rabbits fed a diet containing 0.5% cholesterol was dialyzed against PBS and oxidized by 5 µM copper sulfate (CuSO_4_) for 8 hours at 37°C.

DLH3 or PBS was added to 96-well plates and incubated for 2 hours at room temperature, and then removed from the plates. The plates were incubated with blocking solution for 2 hours at room temperature and then the solution was emptied. Following this, the plates were incubated with solutions of OxLDLs for standard curve generation or LDL dialyzed against 0.25 mM EDTA-4Na (pH 7.4)/PBS of each group for 30 minutes at room temperature and overnight in a refrigerator under protection from light; washed with washing solution; incubated with chicken anti-rabbit apolipoprotein B (apoB) immunoglobulin Y polyclonal antibody (1∶1000 dilution) for 2 hours at room temperature under protection from light; washed with washing solution; incubated with labeled antibody (AP-AffiniPure Donkey Anti-Chicken IgY, Jackson ImmunoResearch Laboratories, Inc, PA, United States) for 2 hours at room temperature under protection from light; washed with washing buffer; rinsed with 50 mM Tris-hydrochloric acid (pH 8.8), and incubated with disodium *p*-nitrophenyl phosphate hexahydrate (1 mg/mL) at room temperature. The absorbance at measuring and reference wavelengths of 405 and 492 nm, respectively, was measured using a plate reader. The OxLDL concentration per well was calculated from the standard curve, and the OxLDL level relative to LDL protein was then calculated by dividing the OxLDL level by the concentration of LDL protein per well.

### Measurement of Oxidation of LDL and HDL

Oxidation of LDL was measured by conjugated diene (CD) formation after lipid peroxidation by 2,2′-azobis(2-methylpropionamidine) dihydrochloride (AAPH) using a UV absorbance detector [23]. Briefly, LDL dialyzed against PBS (100 µg/mL of protein) and 1 mM AAPH were added to 96-well plates, and the accumulation of CD as a result of LDL oxidation at 37°C was measured as the rise in absorbance (at measuring and reference wavelengths of 234 and 205 nm, respectively). To measure the antioxidative effect of HDL on reference LDL isolated from the control group, HDL was added to the reference LDL solution (each at a final concentration of protein at 100 µg/mL). The interval between the start of the reaction and the rise in absorbance (the lag time), maximal rate of oxidation, and maximal concentration of CD were calculated as described previously [24] to evaluate the effects of probucol and atorvastatin on LDL-oxidation.

### Anatomy and Analysis of Atherosclerotic Lesion Area

Six weeks after administration, blood was drawn from the inferior vena cava under pentobarbital anesthesia, and the animals were euthanized by exsanguination. The aorta between aortic arch and the junction of the iliac arteries was then isolated. Pieces were cut from both ends of the thoracic aorta and soaked in cold 4% para-formaldehyde phosphate buffer for histological analysis, and the remaining aorta was soaked in cold saline for en face evaluation of the atherosclerotic lesion area.

The en face evaluation was carried out by carefully removing the adipose and connective tissue surrounding the isolated aorta, cutting open the aorta longitudinally, pinning it to a rubber board with the luminal surface facing up, staining the atherosclerotic lesions of the aorta with Sudan IV, photographing them, and measuring (WinROOF version 5.8.0; Mitani Corporation, Tokyo, Japan) the areas of the atherosclerotic lesions and the area of the whole luminal surface in a blind fashion. The atherosclerotic lesion area relative to the whole luminal surface area (the relative atherosclerotic lesion area) was then calculated for the entire aorta.

For histological analysis, frozen sections were stained with oil red O stain and paraffin sections with Elastica van Gieson stain. Macrophage and α-actin (paraffin sections) and apoB and OxLDL (paraffin and frozen sections) were immunohistochemically stained using RAM11 (Dako Japan Inc., Tokyo, Japan), 1A4 (Dako Japan Inc.), HUC20 (Hiroshima Bio-Medical Co., Ltd., Hiroshima, Japan), and DLH3 [18] antibodies, respectively. Working dilutions of these antibodies were 1∶500, 1∶500, 1∶100, and 1∶1000, respectively.

### Statistical Analysis

Data are presented as the mean ± SD. The add-on effects of probucol when combined with atorvastatin 0.001% (between the 0.001% atorvastatin group and 0.001% atorvastatin +0.1% probucol group) or 0.003% (between the 0.003% atorvastatin group and 0.003% atorvastatin +0.1% probucol group) and the difference in effects of probucol between the control and 0.1% probucol group were analyzed using an unpaired *t*-test. Dose-dependency of the effects of atorvastatin was tested among the control, 0.001% atorvastatin, and 0.003% atorvastatin groups using linear regression analysis, followed by the lower-tailed Williams test when the result was significant or Dunnett’s test when the result was not significant. The effects of probucol and 0.003% atorvastatin were evaluated by comparison to the control group using Dunnett’s test when the 0.001% atorvastatin group and 0.001% atorvastatin +0.1% probucol group were excluded from the analysis. The effects of probucol and/or atorvastatin on the body weight, ALT level, AST level, and CRP concentration were evaluated by comparison to the control group using Dunnett’s test. Differences in the magnitude of the anti-atherosclerotic effects of probucol and atorvastatin in the probucol-treated and probucol-non-treated groups, after elimination of their plasma non-HDLC-lowering effects, were analyzed by two-way analysis of variance (ANOVA). The differences were considered significant at 5% in the two-tailed test. SAS software (Release 9.1; SAS Institute Inc., Tokyo, Japan) was used for all statistical analyses.

## Results

### Plasma Lipids Concentrations

**Figure 1:**
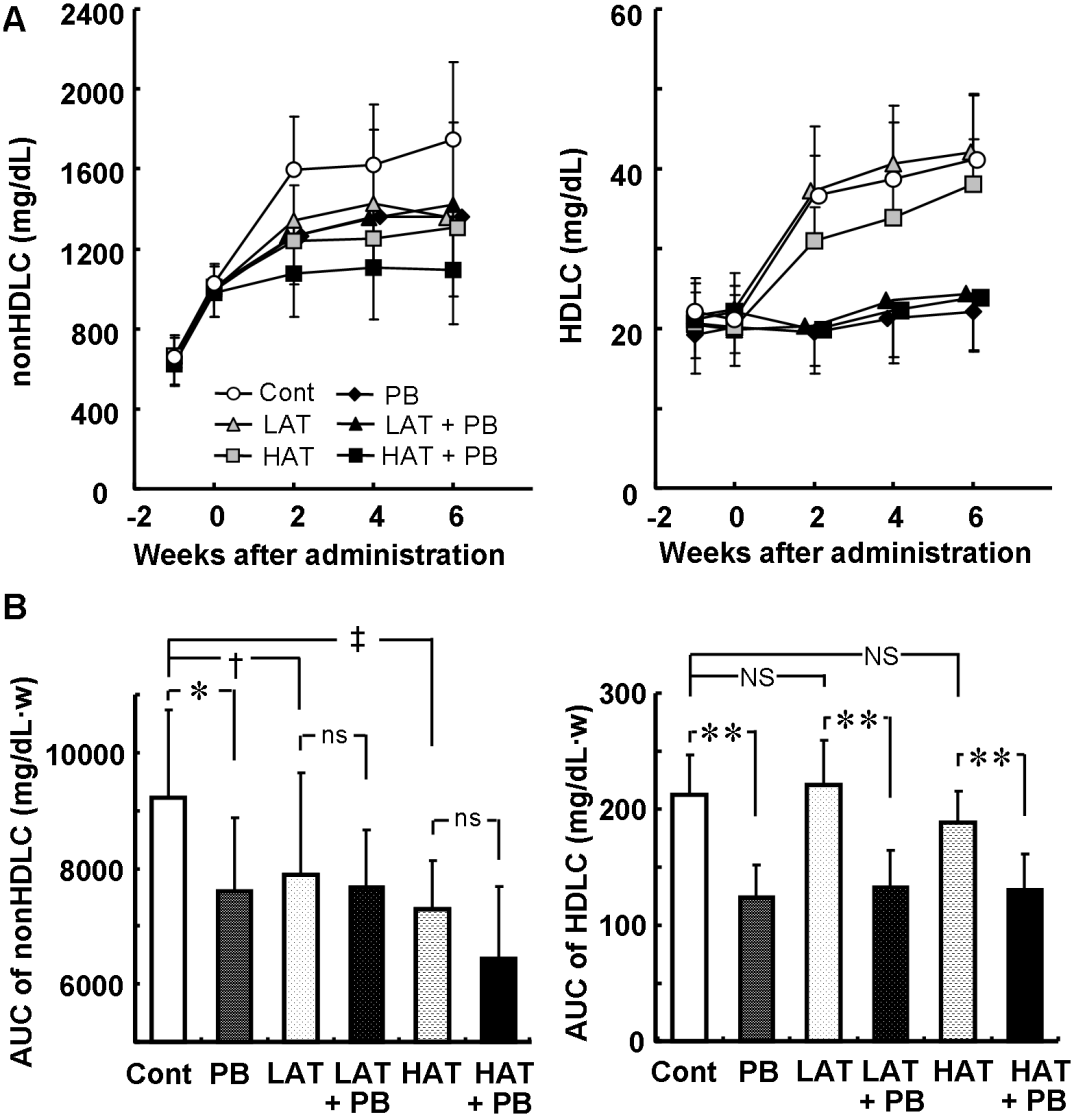
Effects of probucol and atorvastatin on plasma cholesterols. Time-dependent changes in plasma cholesterols (A) and their AUC values over the 6-week period of administration (B). Data are presented as mean ± SD. There were 11 animals in the control (Cont), 0.1% probucol (PB), 0.001% atorvastatin (LAT), LAT + PB groups, and 12 in the 0.003% arorvastatin (HAT) and HAT + PB groups. *p<0.05; **p<0.01; ns, not significant by unpaired t-test. †p<0.05; ‡p<0.01 by Williams test (Regression analysis; linearity, p<0.01; LOF, not significant). NS, not significant by Dunnett’s test.

The effects of probucol and atorvastatin on plasma cholesterols are shown in Figure 1. Plasma concentration of non-HDLC in the control group increased from 1030±85 mg/dL at the start of drug administration to 1750±382 mg/dL at the end of the administration period. Atorvastatin 0.001% and 0.003% dose-dependently decreased the plasma concentration of non-HDLC, and 0.003% atorvastatin decreased the plasma concentration of non-HDLC by 25%, 6 weeks after treatment. Probucol 0.1% also decreased the plasma concentration of non-HDLC to the same extent as atorvastatin (i.e., by 22%); however, when probucol 0.1% was administered in combination with atorvastatin 0.001% or 0.003%, the result was a less pronounced decrease in the plasma concentration of non-HDLC than probucol monotherapy. Probucol with or without atorvastatin inhibited the increase in the plasma concentration of HDLC (Figure 1), while atorvastatin had no effect. Neither probucol nor atorvastatin affected plasma triglyceride concentrations (data not shown).

Thus, both drugs proved to have lipid lowering activity as we predicted in this animal model; however, there were no add-on effects from probucol on the plasma concentration of non-HDLC in combination with atorvastatin.

### Atherosclerotic Lesions

**Figure 2:**
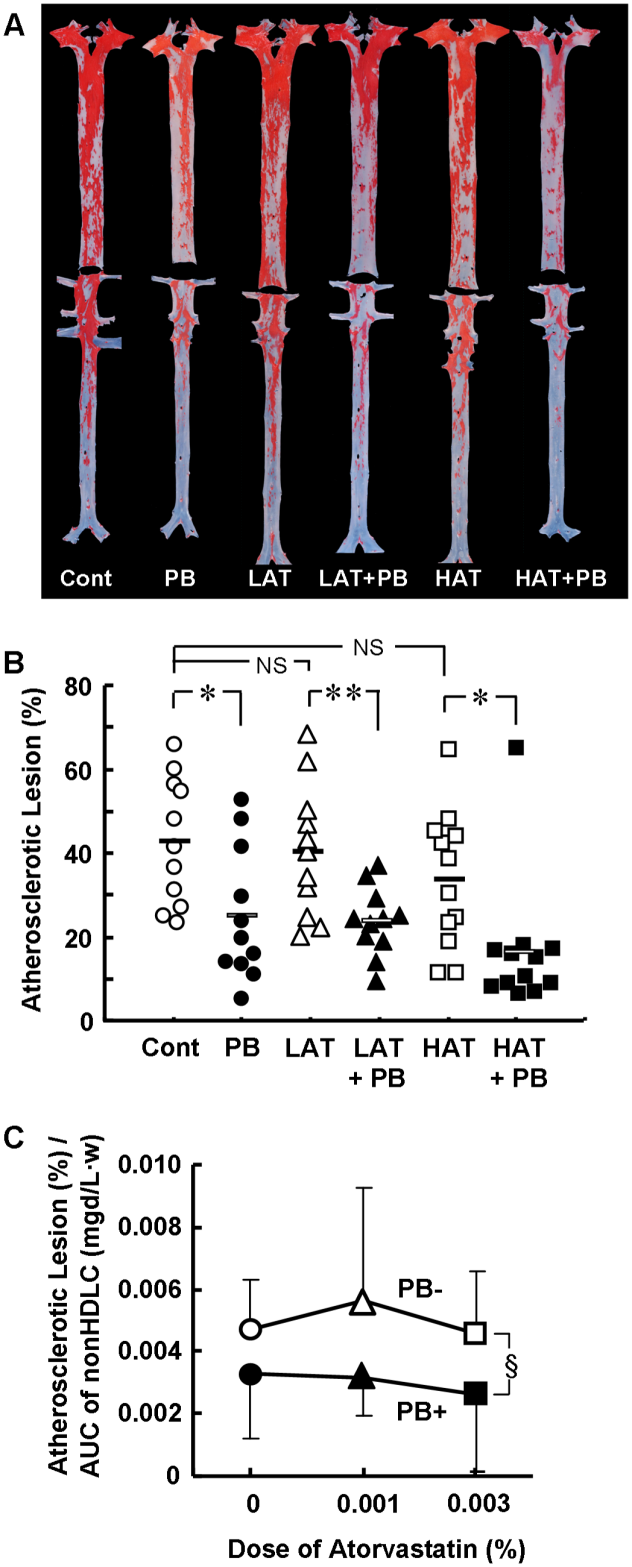
Effects of probucol and atorvastatin on atherosclerotic lesion areas. Representative images of whole atherosclerotic aortas are shown for each group (A). Atherosclerotic lesion areas (ALA) were stained with Sudan IV. Relative ALA of the whole aortas are shown (B). Relative ALA of the whole aorta corrected by the plasma non-HDL cholesterol (nonHDLC) is compared between 0.1% probucol-treated (PB+) and -non-treated (PB−) groups (C). Data are presented as mean ± SD. There were 11 animals in the control (Cont, open circle), PB (closed circle), 0.001% atorvastatin (LAT, open triangle), and LAT+PB (closed triangle) groups, and 12 in the 0.003% atorvastatin (HAT, open square) and HAT+PB (closed square) groups. *p<0.05; **p<0.01 by unpaired t-test. NS, not significant by Dunnett’s test. §p<0.01 by two-way ANOVA.

Effects of probucol and atorvastatin on lesion surface area of the whole aorta are shown in Figures 2A and B. Atorvastatin decreased the lesion area dose-dependently, and the higher dose of atorvastatin reduced the lesion area by 21%; however, these effects were non-significant. The anti-atherogenic effect of atorvastatin at a higher dose (0.005%) was also non-significant [25], suggesting that the anti-atherogenic effects of atorvastatin were limited in this animal model. On the other hand, probucol decreased lesion area by 41%, and this effect of probucol was additive to the anti-atherosclerotic effects of atorvastatin. Probucol plus 0.003% atorvastatin decreased the lesion area by 61%. These anti-atherosclerotic effects of probucol were similar in both the thoracic and abdominal aorta (data not shown).

The potent anti-atherosclerotic effects of probucol could not be attributed solely to its plasma non-HDLC-lowering effects. Therefore, to confirm the contribution of effects other than the non-HDLC-lowering effect, the surface areas of atherosclerotic lesions were divided by the plasma non-HDLC concentrations, and the corrected values for the probucol-treated and non-probucol-treated groups were compared. The corrected values were found significantly smaller in probucol-treated groups than in non-probucol-treated groups (Figure 2C), indicating that part of the anti-atherosclerotic effect of probucol could be due to an effect that is distinct from lowering of the plasma concentration of non-HDLC.

**Figure 3:**
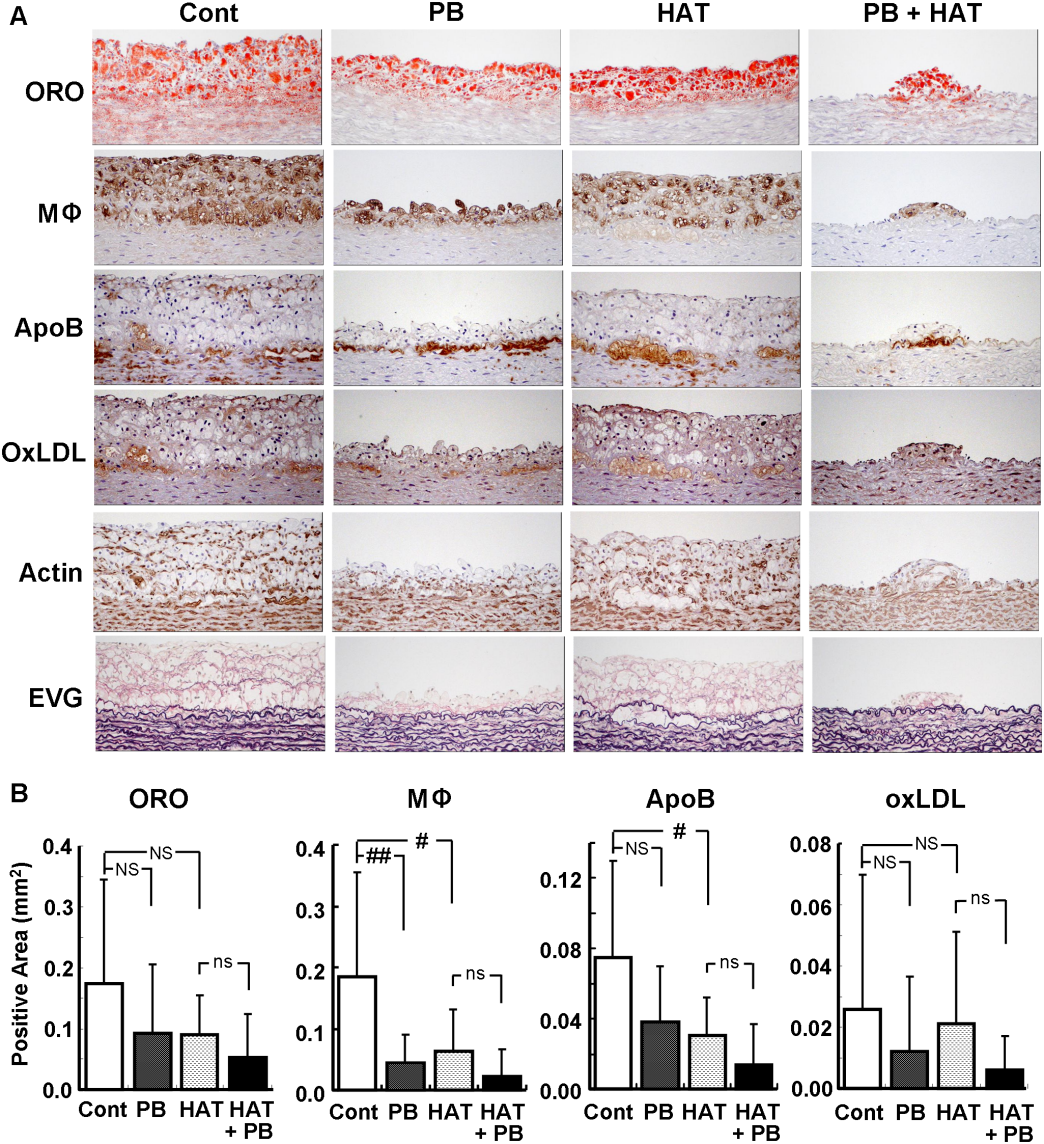
Histological analysis of an atherosclerotic lesion in the thoracic aorta. Representative histological images of an atherosclerotic lesion in the thoracic aorta of each group are shown (A). Quantitative analysis of the Oil red O (ORO)-, macrophage (Mφ)-, apolipoprotein B (ApoB)-, and OxLDL-positive areas are indicated (B). Staining with ORO and other reagents was performed using frozen and paraffin sections, respectively. Data are presented as mean ± SD. There were 11 animals in the control (Cont) and 0.1% probucol (PB) groups, and 12 in the 0.003% atorvastatin (HAT) and HAT + PB groups. #p<0.05; ##p<0.01; NS, not significant by Dunnett’s test. ns, not significant by unpaired t-test. Actin, α-actin; EVG, Elastica van Gieson.

To examine the mechanisms of the anti-atherosclerotic activity of these drugs, atherosclerotic lesions of the thoracic aorta were analyzed histologically. Histological analysis using paraffin sections revealed that the atherosclerotic lesions contained mainly macrophages, and apoB and OxLDL were intensively stained at the boundary of the intima and media (Figure 3A). The analysis using frozen sections revealed apoB- and OxLDL-stained areas throughout the plaque (data not shown). Both probucol and atorvastatin reduced the accumulation of macrophages by 76% and 65%, respectively, and oil red O-stained areas by half. The accumulation of apoB was reduced to a slightly greater extent by atorvastatin than by probucol, while the accumulation of OxLDL was reduced by 54% by probucol but only slightly by atorvastatin. A combination of these drugs reduced accumulation more effectively than monotherapy with either drug (Figure 3B). These results suggest that different mechanisms underlie the anti-atherosclerotic effects of these drugs, and that part of the anti-atherosclerotic effect of probucol could be due to its antioxidative effect.

### Antioxidative Effects

**Figure 4:**
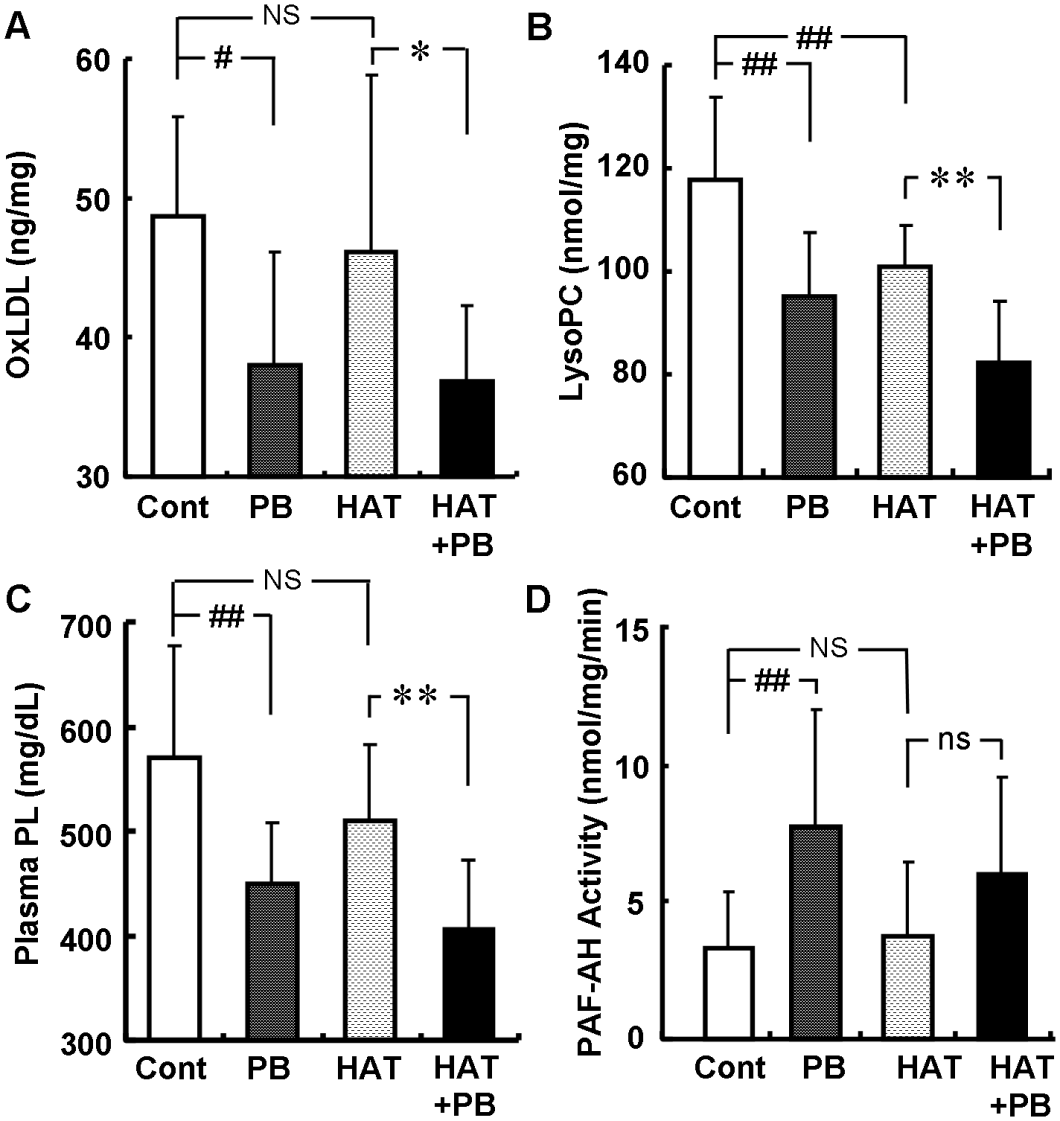
Anti-oxidative Effects of probucol and atorvastatin on plasma lipids. Effects on OxPC (A) and LysoPC (B) contents in LDL, plasma phospholipids concentration (C) and PAF-AH activity in LDL (D) at 6 weeks after administration. Data are presented as mean ± SD. There were 10 or 11 animals in the control (Cont) and 0.1% probucol (PB) groups, and 12 in the 0.003% atorvastatin (HAT) and HAT + PB groups. *p<0.05; **p<0.01; ns, not significant by unpaired t-test. #p<0.05; ##p<0.01; NS, not significant by Dunnett’s test.

To examine the antioxidative effects of probucol and atorvastatin, we measured plasma levels of oxidized lipids. Probucol significantly decreased the plasma OxLDL level measured by a modified sandwich ELISA using anti-oxidized phosphatidylcholine monoclonal antibodies and anti-apoB polyclonal antibodies [21] (Figure 4A). Probucol also decreased LysoPC content in LDL (Figure 4B). The extent of these decreases was similar regardless of whether probucol was administered alone or with atorvastatin. On the other hand, 0.003% atorvastatin decreased LysoPC content in LDL but did not affect the plasma OxLDL level (Figures 4A and B). Probucol inhibited the increase in plasma phospholipid concentration (Figure 4C) and increased the activity of PAF-AH in LDL (Figure 4D), while atorvastatin did not.

**Figure 5:**
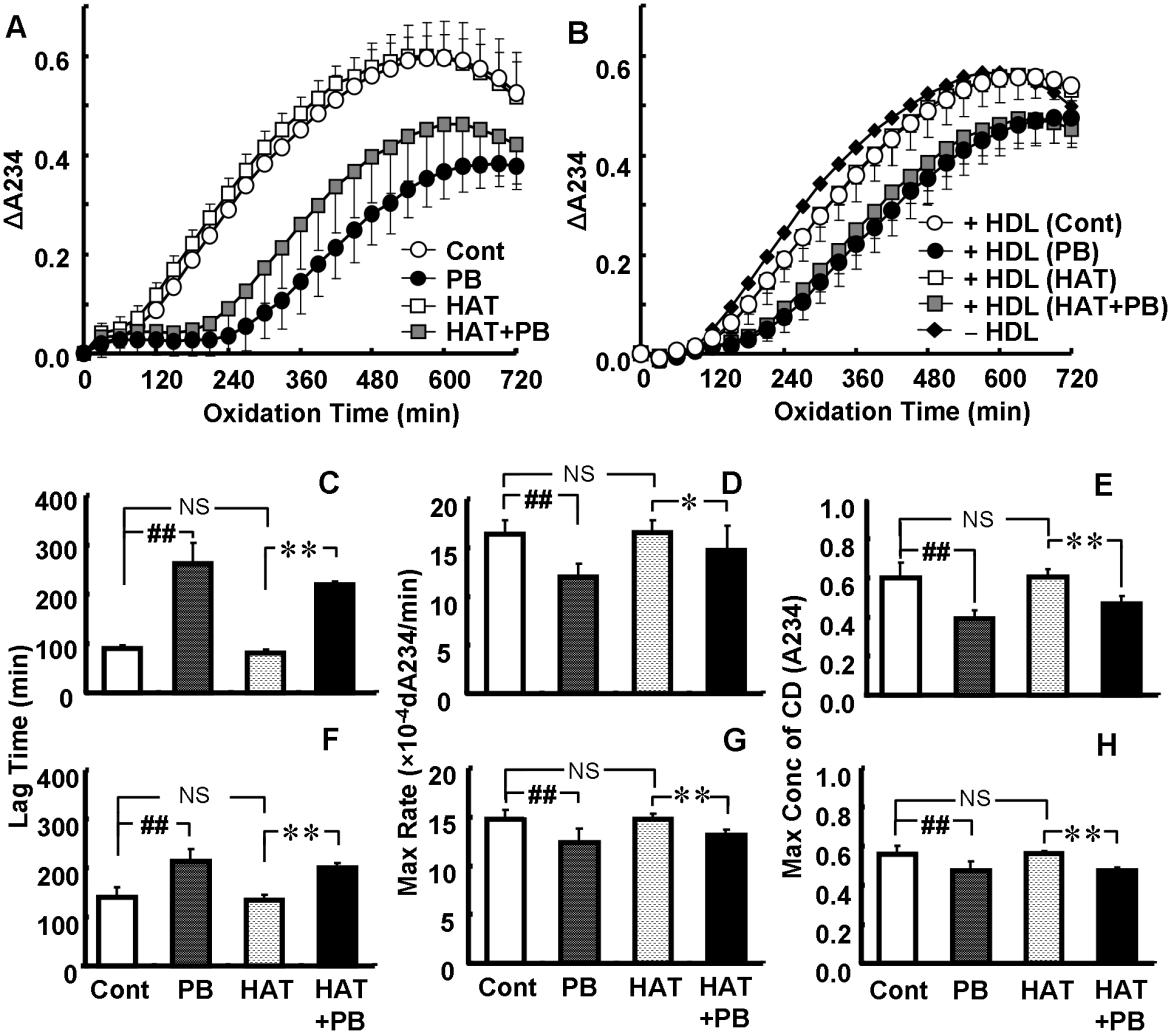
Protective Effects of probucol on LDL-oxidation. Effects on LDL-oxidation (A, C, D, E) and anti-oxidative effects of HDL on LDL-oxidation (B, F, G, H). LDL was oxidized by AAPH. Pooled LDL from the control group was used as reference LDL in the experiment examining the anti-oxidative effects of HDL. Data are presented as mean ± SD. There were 11 animals in the control (Cont) and 0.1% probucol (PB) groups, and 12 in the 0.003% atorvastatin (HAT) and HAT + PB groups. The lines of HDL (HAT) and HDL (HAT+PB) are overlaid by HDL (Cont) and HDL (PB), respectively (B). *p<0.05; **p<0.01 by unpaired t-test. ##p<0.01; NS, not significant by Dunnett’s test. Max Rate, maximum rate of oxidation; Max Conc of CD, maximum concentration of conjugated diene.

An in vitro LDL-oxidation study revealed that LDLs from probucol-treated rabbits were significantly resistant to oxidation induced by AAPH (Figures 5A, C, D, and E) or copper sulfate (CuSO_4_) (data not shown). In contrast, LDLs from atorvastatin-treated rabbits did not show any such antioxidative effects. Resistance to oxidation of LDLs from probucol plus atorvastatin-treated rabbits was also shown. HDL from probucol-treated rabbits had enhanced ability to protect against oxidation by AAPH (data not shown), and significantly inhibited the oxidation of reference LDL by AAPH (Figures 5B, F, G, and H). HDLs from atorvastatin-treated rabbits had no such antioxidative effects.

**Figure 6:**
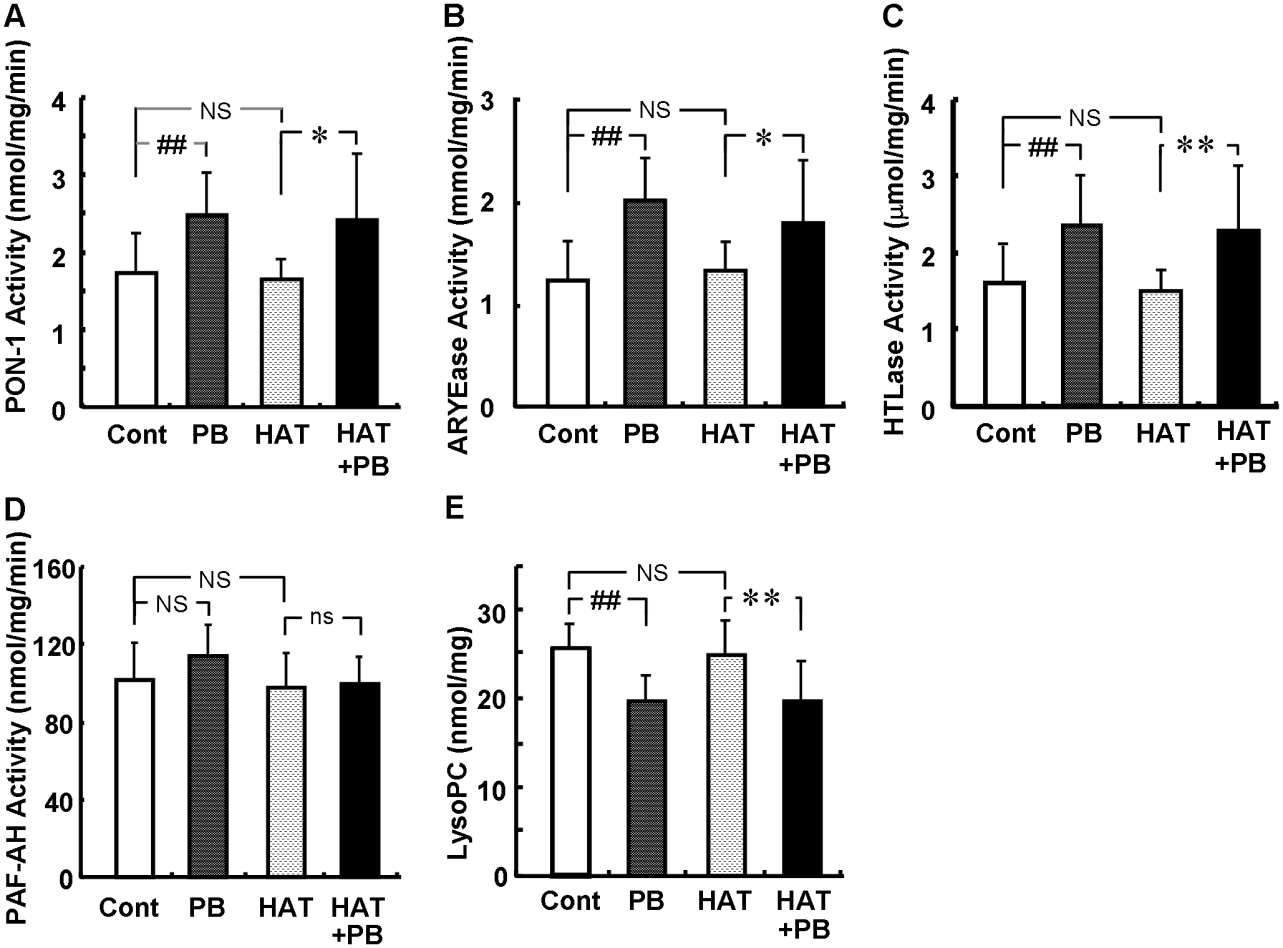
Effects of probucol on anti-oxidative enzyme activities in HDL. Effects on PON-1 (A), arylesterase (ARYEase) (B), homocysteine thiolactonase (HTLase) (C) and PAF-AH (D) activities, and LysoPC contents (E) in HDL at 6 weeks after administration. Data are presented as mean ± SD. There were 11 animals in the control (Cont) and 0.1% probucol (PB) groups, and 12 in the 0.003% atorvastatin (HAT) and HAT + PB groups. *p<0.05; **p<0.01 by unpaired t-test. ##p<0.01; NS, not significant by Dunnett’s test.

Probucol increased PON-1 activities, tended to increase PAF-AH activity and decreased LysoPC contents in HDL, while atorvastatin did not (Figure 6).

### Plasma C-reactive Protein and General Conditions

Plasma CRP concentrations of rabbits in the control group fed a high-cholesterol diet for eight weeks (3.8±1.9 µg/mL) were within the range of concentrations in rabbits fed a normal diet [26]. Neither probucol nor atorvastatin influenced the plasma CRP concentration (Table 1). Increase in the level of liver damage markers after atorvastatin treatment is a common adverse effect of atorvastatin. Atorvastatin 0.003% but not 0.001% doubled plasma ALT and AST concentrations (Table 1), suggesting that the doses we used are equivalent to the maximum dose prescribed clinically. In contrast, probucol had no effect on the levels of these liver damage markers (Table 1). Furthermore, the plasma probucol concentrations (11–14 µg/mL) in our rabbits 6 weeks after treatment were much lower than those (31–60 µg/mL) in patients receiving probucol (500 mg twice daily) for treatment of familial hypercholesterolemia [7]. Body weights of rabbits in all 6 groups remained almost the same throughout the experiment (Table 1) and the general condition of rabbits in all 6 groups remained normal.

**Table 1:** Body Weight and Plasma Parameters.

	Control	PB	LAT	LAT+PB	HAT	HAT+PB
BW (g)	2422±103	2422±106^NS^	2449±81^NS^	2465±129^NS^	2435±128^NS^	2401±93^NS^
ALT (IU/L)	59±14	51±13^NS^	66±21^NS^	74±22^NS^	143±101†† ^,^ ##	128±30##
AST (IU/L)	15±4	14±3^NS^	15±7^NS^	16±4^NS^	35±15##	48±28##
CRP (µg/mL)	3.8±1.9	3.0±2.1^NS^	5.3±1.8^NS^	4.5±2.7^NS^	4.5±2.6^NS^	3.8±1.9^NS^
PB (µg/mL)	–	13.9±5.7	–	12.7±4.2	–	11.1±3.3
n	11	11	11	11	12	12

Values at 6 weeks after administration are presented as mean ± SD. BW, body weight; ALT, alanine aminotransferase; AST, aspartate aminotransferase; CRP, C-reactive protein; PB, plasma probucol concentration.

†† p<0.01 by Williams test (Regression analysis; linearity was significant, lack of fit [LOF] was not significant).

## p<0.01; NS, not significant by Dunnett’s test. There are no significant differences between control and 0.1% probucol (PB) groups, 0.001% atorvastatin (LAT) and LAT+PB groups, and 0.003% atorvastatin (HAT) and HAT+PB groups by t-test.

## Discussion

In this study, it was clearly shown that probucol has a potent effect that was additive to that of atorvastatin when administered to atherosclerotic rabbits fed a 0.5% cholesterol diet. Probucol 0.1% with 0.003% atorvastatin was shown to have a strong anti-atherosclerotic effect, with a 61% reduction in lesion formation in animals that already had plasma non-HDLC high enough to induce atherosclerotic lesion formation. The dose of probucol (0.1%; equivalent to 31–50 mg/kg/day) is lower than that used in other rabbit studies on atherosclerosis [8], [17], and the plasma probucol concentrations (11–14 µg/mL) 6 weeks after treatment were much lower than those (31–60 µg/mL) used in patients receiving probucol (500 mg twice daily) treatment for familial hypercholesterolemia [7]; however, 0.1% probucol significantly decreased the area of atherosclerotic lesions by 41%. On the other hand, the doses of atorvastatin used in this study decreased plasma concentration of non-HDLC, but a higher dose (0.003%) slightly increased the levels of liver damage markers, and the area of atherosclerotic lesions were decreased by just 21%, suggesting that the anti-atherosclerotic effects of atorvastatin were limited in this animal model.

One possible mechanism by which a combination of drugs inhibits atherogenesis is lowering of the plasma non-HDLC concentration. Statins lower plasma LDL cholesterol markedly by inhibiting HMG-CoA reductase, resulting in up-regulation of LDL receptor expression, and reduce VLDL production in the liver. On the other hand, probucol lowers plasma concentration of LDL cholesterol by up-regulating the fractional catabolic rate of LDL via LDL receptor-independent pathway [27] and reverse cholesterol transport by increasing bile acid synthesis [28]. Therefore, it is expected that probucol combined with atorvastatin will have an add-on effect to lower plasma concentration of non-HDLC. However, the add-on effects of probucol on plasma concentration of non-HDLC compared with those of atorvastatin, were not significant in this study and seem to be insufficient to explain the potent add-on effect of probucol in preventing atherosclerotic lesion formation.

Probucol inhibited an increase in plasma concentration of HDLC but enhanced the activity of PON-1, an enzyme that protects HDL from oxidation [29], as was found in another study [24], suggesting that the inhibitory effect of probucol on increases in plasma concentration of HDLC did not act as a proatherogenic factor in this animal model.

Another possible mechanism is that probucol acts as a strong antioxidant. OxLDL consists of several heterogeneously modified phospholipids (e.g., OxPC, LysoPC) and low molecular weight aldehydes (e.g., malondialdehyde); it is considered to be the main lipoprotein involved in initiation and development of atherosclerosis and an important marker of cardiovascular disease [6], [30]. Unlike statins, probucol has a diphenol moiety with potent free radical-scavenging activity and significantly protects LDL from oxidation by AAPH as previously reported [7]. In addition, probucol, unlike other phenolic antioxidants such as vitamin E, enhanced the protective effect of HDL on LDL oxidation by enhancing the PON-1 activities of HDL, as previously reported [24]. Probucol is also reported to increase the activity of heme oxygenase-1, a redox-sensitive enzyme responsive to several stress stimulants such as OxLDL [31], and to decrease the expression of NAD(P)H oxidase [32] in aortic smooth muscle cells in rabbits. Such antioxidative effects of probucol might be more important than its free radical scavenging activity in terms of the prevention of atherosclerosis. Although statins are also reported to have such anti-oxidative effects (i.e., an increase in heme oxygenase-1 expression and a decrease in NAD(P)H oxidase activity [19]), probucol but not atorvastatin decreased the plasma and plaque OxLDL levels, indicating the antioxidative effect of probucol differs in vivo from that of atorvastatin. Previous work found that plasma OxLDL levels in apolipoprotein E-knockout mice measured by our sandwich ELISA procedure preceded the formation of extensive atherosclerotic lesions, suggesting that OxLDL has pro-atherosclerotic properties in vivo [22]. Therefore, the probucol-induced decrease in the plasma OxLDL level seems to be related to its anti-atherosclerotic effect. On the other hand, both probucol and atorvastatin decreased LysoPC content in LDL, although probucol increased the PAF-AH activity of LDL while atorvastatin did not. Recognized as an important cell signaling molecule, LysoPC is produced by the action of phospholipase A_2_ (including PAF-AH) on PC and OxPC, and is implicated as a critical factor in atherogenesis as it stimulates superoxide anion production via endothelial NAD(P)H oxidase [33]. Hence, LysoPC plays an important role in atherosclerosis [34]. PAF-AH can also degrade not only the potent inflammatory mediator PAF but also PC analogues that contain a hydrophilic short chain moiety at the sn-2 position, such as OxPCs. Therefore, whether PAF-AH plays a protective role in atherogenesis by hydrolyzing OxPC and PAF, or rather acts as a proatherogenic factor by releasing LysoPC remains controversial. However, probucol decreased plasma LysoPC concentration irrespective of an increased effect on PAF-AH activity. This could be a result of its protective effects on LDL oxidation and an effect that lowers plasma phospholipid concentration. The ability of probucol to reduce the OxPC level and increase PAF-AH activity in LDL, which are not inherent properties of atorvastatin, might explain the different potential these drugs have for anti-atherosclerotic effects and the significant add-on effect of probucol on atherogenesis in this animal model when probucol was combined with atorvastatin.

These drugs also have various antiinflammatory activities [13], [19]. Plasma CRP concentration is a clinically important marker of increased risk of cardiovascular disease, and CRP concentration in rabbits is increased by feeding a cholesterol-enriched diet for longer than 12 weeks [26]. However, plasma CRP concentration in this experimental period (during the 8-week period of feeding with a diet containing 0.5% cholesterol) remained within the normal range and was not affected by either drug. Therefore, inflammation accompanied by increasing plasma CRP concentration is not associated with atherogenesis in this rabbit model, and the antiinflammatory effects of these drugs need to be examined at a higher plasma CRP concentration, similar to that seen in the patients with atherosclerosis. Meanwhile, consumption of a cholesterol-enriched diet significantly induced (and both drugs significantly inhibited) macrophage accumulation in the sub-endothelial layers. Therefore, we need to investigate other antiinflammatory effects and the pleiotropic effects of these drugs that lead to the inhibition of monocyte accumulation and foam cell formation such as inhibitory effects on the expression of endothelial adhesion molecules or secretion of monocyte chemotactic protein-1.

While probucol attenuates atherosclerotic lesion development in most animal models [8]–[10], it promotes atherosclerosis in apolipoprotein E-deficient mice [11], [35], [36]. Increased plasma fibrinogen [35] or alterations in the immune system [36] after probucol treatment may be involved in the proatherogenic effects of probucol; however, the exact mechanisms enhancing atherosclerosis in mice are currently unknown. No reports of such proatherogenic effects are reported in humans.

In this study, probucol showed significant additive anti-atherosclerotic effects compared to the limited anti-atherosclerotic effect of atorvastatin, probably due to its potent antioxidative activity that is unrelated to the pleiotropic effects of atorvastatin. Moreover, a lower dose of probucol resulted in marked anti-atherosclerotic effects, suggesting that probucol at doses lower than those currently used clinically could be effective in the treatment of patients with atherosclerosis. These results strongly suggest that administering a combination of probucol and statins would be a good strategy for treating atherosclerotic disorders in patients who have an inadequate response to statins. Further investigation on conditions accompanying atherosclerosis such as diabetes or obesity is needed to clarify clinical efficacy of this combination in preventing or treating atherosclerotic disorders.
